# Delayed gastropleural fistula: a rare cause of a persistent pleural effusion after blunt force trauma

**DOI:** 10.1093/jscr/rjab117

**Published:** 2021-04-20

**Authors:** Kin Yik Chan, Shane Keogh, Nitin Aucharaz, Hugo Temperley, James O’Driscoll, Narayanasamy Ravi, Shona Tormey

**Affiliations:** University Hospital Limerick, Department of Breast and General Surgery, Dooradoyle, Limerick, Ireland; University Hospital Limerick, Department of Breast and General Surgery, Dooradoyle, Limerick, Ireland; University Hospital Limerick, Department of Breast and General Surgery, Dooradoyle, Limerick, Ireland; St.James's Hospital, Department of General Surgery, D08 NHY1, Dublin, Ireland; University Hospital Limerick, Department of Anaesthetics and Critical Care, Dooradoyle, Limerick, Ireland; St.James's Hospital, Department of General Surgery, D08 NHY1, Dublin, Ireland; University Hospital Limerick, Department of Breast and General Surgery, Dooradoyle, Limerick, Ireland

## Abstract

A gastropleural fistula (GPF) is a rare pathological connection between the stomach and pleural cavity. Diagnosis and treatment are frequently delayed due to the lack of specific clinical, laboratory and radiological findings. We describe a case of a 53-year-old gentleman who presented to our institution with respiratory sepsis and a massive haemopneumothorax on imaging. Uniquely, he was discharged a week prior after a splenectomy for a traumatic fall. Gut flora in the pleural fluid and a subsequent positive dye test suggested an aero-digestive connection. Repeat imaging revealed a fistula between stomach and the left pleural cavity through a ruptured diaphragm. He underwent an open sleeve gastrectomy and primary repair of the diaphragm. This is the first GPF in literature presenting in such a fashion. Although rare, a persistent effusion with a history of blunt thoracoabdominal trauma may herald a GPF, which, if not diagnosed promptly, may result in significant morbidity.

## INTRODUCTION

Gastropleural fistulas (GPF) are rare pathological connections between the stomach and pleural cavity. Mechanistically, Markowttz [1] first described the trans-diaphragmatic extension of an inflammatory response, from a perforated hiatal hernia or gastric ulcer, into the pleural cavity. There has been an increasing incidence in parallel with increasing volume of bariatric surgeries [2]. The unifying aetiology involves instrumentation and inflammation in close proximity to the diaphragm. While GPF as a consequence of penetrating trauma has been described [3], blunt force trauma is an extremely rare cause of a GPF, last being reported in 1947 [1] and 1952 [1], respectively. We report a unique case of a delayed GPF after blunt thoracoabdominal trauma.

## CASE REPORT

A 53-year-old gentleman was admitted after falling down a flight of stairs, landing on his left torso. Imagining identified left rib 10–12 fractures, flail chest, a left haemopneumothorax and an AAST grade-IV splenic laceration. A chest drain was inserted, and he underwent an emergency laparotomy and splenectomy with no intraoperative complications. His chest drain was removed on post-operative day three, and he was discharged on post-operative day seven after an uneventful recovery.

**Figure 1:**
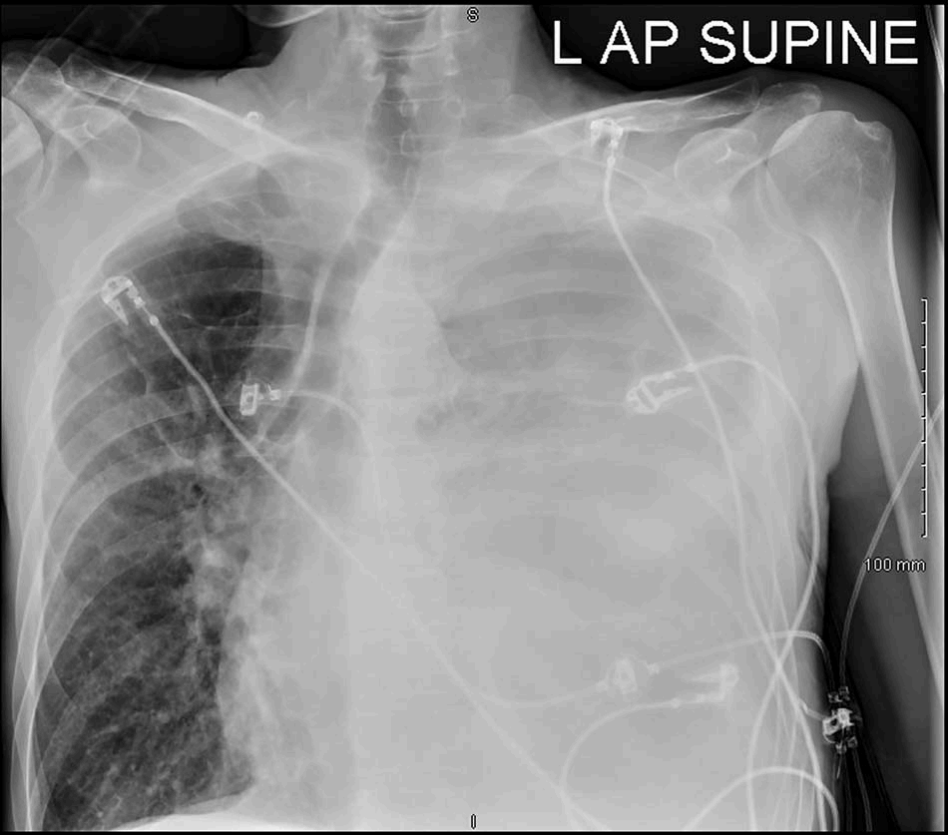
Complete opacification of left hemithorax with mass effect.

**Figure 2:**
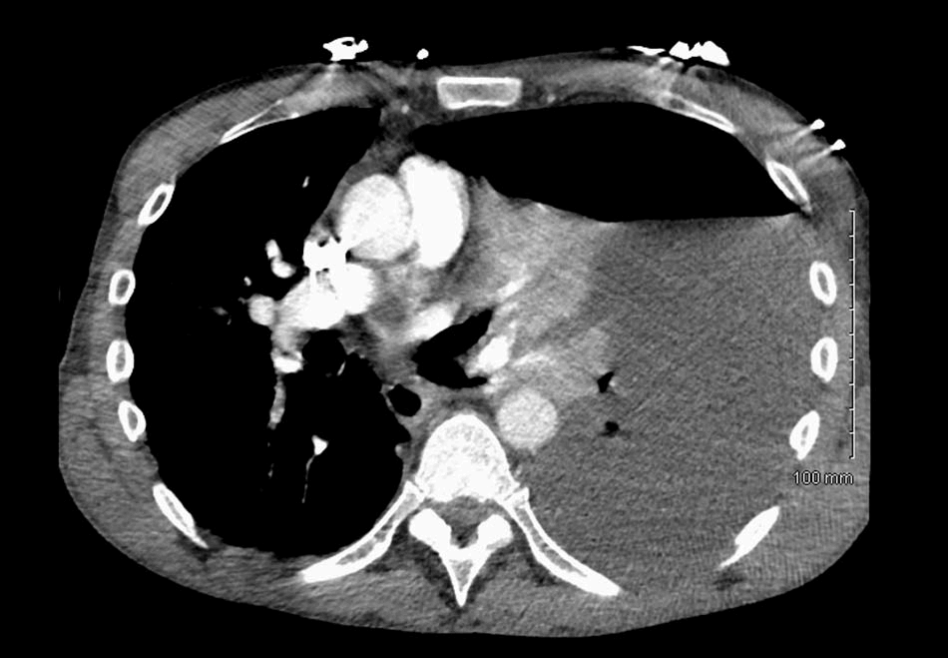
Massive haemopneumothorax on CT.

He was readmitted three days post-discharge with worsening pleuritic chest pain and dyspnoea. On admission, he was febrile and tachycardic. On examination, he had absent breath sounds and dullness to percussion over the left mid and lower zones of his chest. Chest x-ray confirmed a large left-sided pleural effusion (Fig. 1), which was consistent with a massive haemopneumothorax on cross-sectional imaging (CT) (Fig. 2). A chest drain was inserted that drained 6 l of serosanguinous fluid. The patient deteriorated thereafter and required inotropic support and empirical broad-spectrum antimicrobials, piperacillin-tazobactam (4.5 g IV) and gentamicin (5 mg/kg IV) in the intensive care unit.

**Figure 3:**
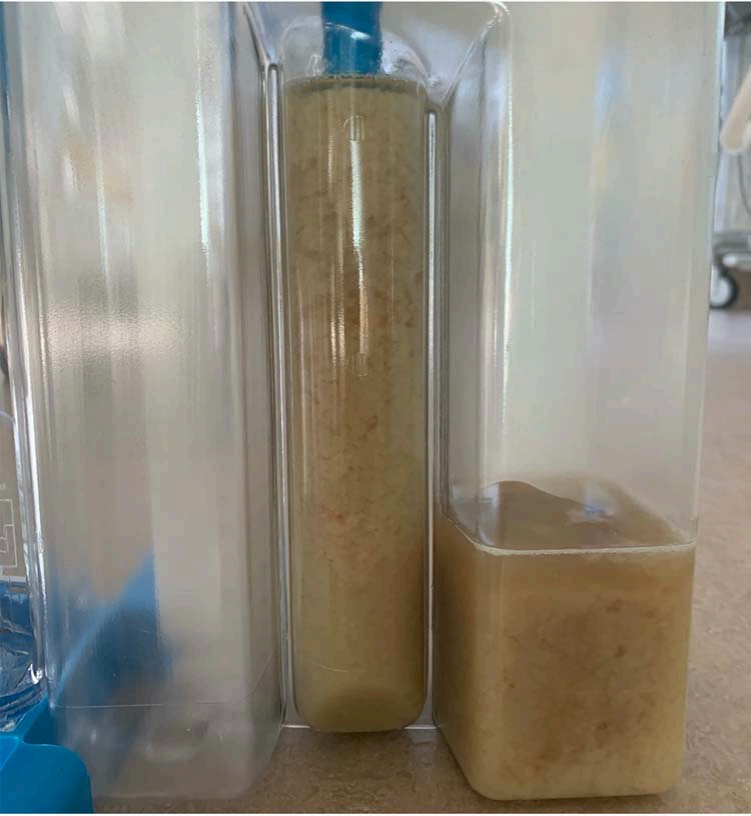
Increased sedimentation post enteral feeding.

**Figure 4:**
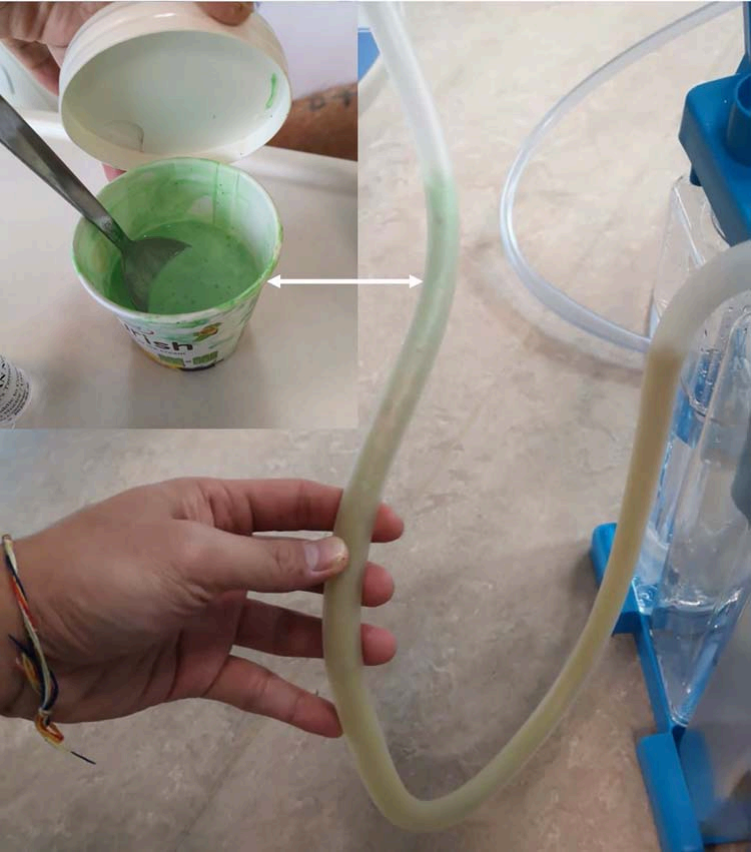
Positive dye test with green ice cream.

**Figure 5:**
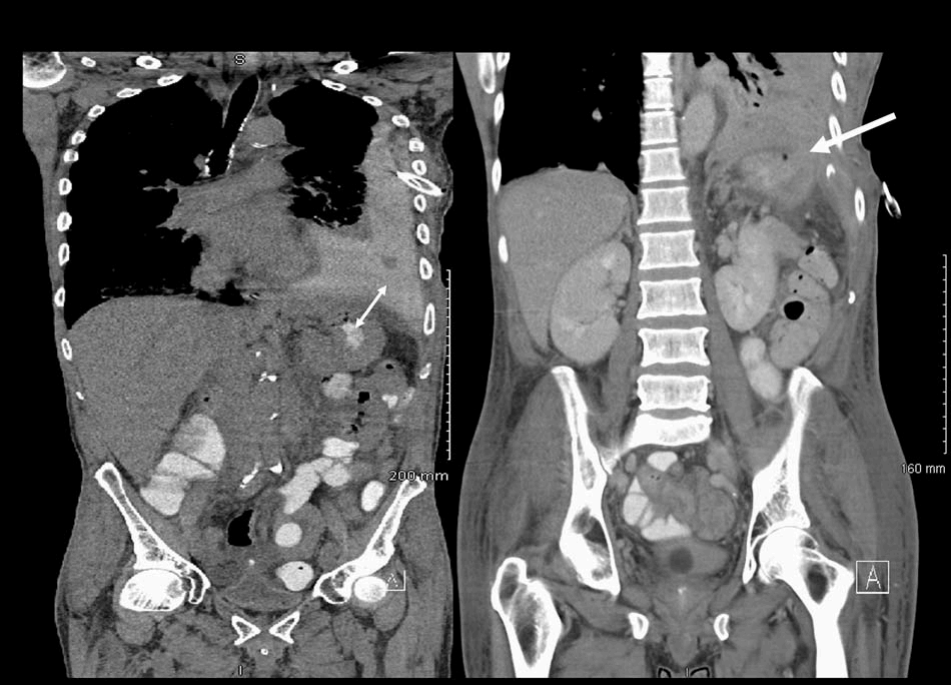
(Left) Contrast in stomach and pleural fluid are iso-dense confirming a fistula; (Right) neck of hernia in the diaphragm.

He maintained a high daily chest drain output of between 2.5 and 5 l. Upon recommencement of enteral feeding on day 3, we noticed an increase in opacity of the drainage fluid (Fig. 3). Normal triglyceride and chylomicron levels ruled out a chylothorax. Cultures from the drain eventually grew *Enterococcus faecium*, *Candida albicans* and *Escherichia coli* consistent with gut flora. At this point, an aerodigestive fistula was suspected. This was confirmed by a dye test subsequently performed with green ice cream, which tainted the drainage fluid green (Fig. 4). A repeat CT scan with oral and intravenous contrast delineated a fistula between the greater curvature of the stomach and the left basal pleura, with an extensive empyema (Fig. 5).

The patient was transferred to a tertiary upper-gastrointestinal surgical unit on day 5 of his admission. After an unsuccessful attempt at endoscopic fistula occlusion, he underwent an emergency laparoscopic hernia reduction and sleeve gastrectomy to obliterate the fistula. The large amount of pus and solid food debris necessitated a conversion to open laparotomy for a thorough pleural lavage. Chest drains were inserted and secured towards the base of the left pleural cavity. The diaphragmatic defect was repaired with interrupted 1–0 Prolene sutures. A thorough washout of the abdominal cavity was performed with placement of a drain in the subdiaphragmatic space before closure. A separate decortication procedure of the left lung was required to facilitate re-expansion. After a prolonged hospital stay for rehabilitation, the patient was discharged home day 24 postoperatively and has remained well.

## DISCUSSION

GPFs secondary to blunt force trauma are extremely rare. Traumatic GPFs are often associated with underlying diaphragmatic ruptures, which occur in up to 5% of blunt thoracoabdominal injuries [4]. Up to 30% of diaphragmatic injuries manifest late [4] due to the evolving necrosis and progressive loss of diaphragmatic integrity with time [4]. Our patient presented 10 days after his initial trauma, corroborating the difficulties of an early diagnosis.

Our patient presented in advanced stages of sepsis secondary to his empyema. Other symptoms that have been reported in the literature include haemoptysis, purulent cough, recurrent pneumonia, shoulder tip and abdominal pain [2]. Given the masquerading presentations, a high index of suspicion is essential. Unfortunately, routine laboratory tests are not specific for GPFs. The presence of gut flora in the pleural fluid may suggest an aerodigestive connection. Additionally, we found the dye test [1] to be sensitive in confirming our suspicions prior to further sophisticated imaging.

There are no specific signs of GPF on a plain film. Surrogate signs of a diaphragmatic defect include an intrathoracic stomach or nasogastric tube and elevation of the hemidiaphragm [5]. Notably, up to 66% of diaphragmatic injuries are missed on a plain film [5]. Although CT has a specificity of up to 99% for diagnosing diaphragmatic defects [5], it is again not specific for GPFs. Based on our institutional experience, the diagnostic yield for a GPF can be increased with modifications to CT protocols. The administration of both oral and intravenous contrast allowed visualisation of contrast extravasation from the stomach into the pleural cavity. Clamping of the chest drain and keeping the patient supine for an hour after consumption of oral contrast ensured that there was maximal accumulation of contrast in the pleural cavity. The gold standard for diagnosis remains direct intra-operative visualisation [2].

Given the rarity of GPF’s, there is no defined standard of care. Early source control in this case with thoracostomy and commencement of broad-spectrum antimicrobials mitigated significant morbidity. Urgent surgery is required for definitive fistula obliteration. Published approaches include endoscopic techniques for fistula occlusion (e.g. fistula clipping, argon coagulation, fibrin glue, mesh application) [2, 6, 7], direct laparoscopic repair [2, 8], and open repair using either an abdominal [2] or thoracic approach [9]. Classically an open approach via laparotomy was preferred, with a thoracotomy in cases with high-volume thoracic contamination [2]. Laparoscopic approaches are controversial in management of co-located abdominal and thoracic injuries owing to the lack of appropriated access to both anatomical fields [2]. Ultimately, the choice of technique is guided by surgeon preference. Current evidence does not support the use of conservative management [6]. Despite complex management strategies, the morbidity associated with GPF is high.

## CONCLUSION

This is the first case in literature reporting a GPF presenting in such a fashion. Thorough assessment and decisive action led to a satisfactory outcome. Although rare, a persistent effusion with a history of blunt thoracoabdominal trauma may herald a GPF, which, if not diagnosed promptly, may result in significant morbidity.

## CONFLICT OF INTEREST STATEMENT

There is no conflict of interest to report.

## FUNDING

The authors received no funding for the research, authorship and publication of this article.

## References

[1] Markowttz A, Herter F. Gastro-pleural fistula as a complication of esophageal hiatal hernia^*^. Ann Surg 1960;152:129–34. 14421290 10.1097/00000658-196007000-00018 PMC1613612

[2] Alghanim F, Alkhaibary A, Alzakari A, AlRumaih A. Gastropleural fistula as a rare complication of gastric sleeve surgery: a case report and comprehensive literature review. Case Reports in Surgery 2018;2018:1–5. 10.1155/2018/2416915 PMC6317118 30671274

[3] Ayyat KS . Stomach tear through chest making an awkward connection (traumatic gastro-pleural fistula). MOJ Clinical & Medical Case Reports 2016;4:131–3.

[4] Rashid F, Chakrabarty MM, Singh R, Iftikhar SY. A review on delayed presentation of diaphragmatic rupture. World J Emer Surgery 2009;4:32. 10.1186/1749-7922-4-32 PMC2739847 19698091

[5] Iochum S, Ludig T, Walter F, Sebbag H, Grosdidier G, Blum AG. Imaging of diaphragmatic injury: a diagnostic challenge? Radio Graphics 2002;22:S103–16. 10.1148/radiographics.22.suppl_1.g02oc14s103 12376604

[6] Baka N, Batra V, Yeung V, Diagnosis LS. Management of Gastro-pleural Fistula in metastatic malignancy. Cureus 2019;11:e4455. 10.7759/cureus.4455 PMC6561524 31205841

[7] Andrawes S, El Douaihy Y. Using the endoscopic overstitching device and fully covered esophageal stents for closure of a gastropleural fistula and repair of a deformed gastric sleeve. Video GIE 2017;2:98–9. 29905278 10.1016/j.vgie.2017.02.003 PMC5990991

[8] Luo RB, Liu S, DeLong JC, Jacobsen GR, Sandler BJ, Horgan S. Laparoscopic Gastropleural fistula repair: a minimally invasive solution for a complex problem. Videoscopy 2017;27(4). 10.1089/lap.2016.0569 28080207

[9] Kathayanatt JT, Palangadan S, Radhakrishnan R, Thanath J. An Unusual Case of Gastropleural Fistula: Management Dilemmas. Lung India: Official Organ of Indian, 2020. 10.4103/lungindia.lungindia_242_17 PMC7065535 32108607

